# Specific exercise training for reducing neck and shoulder pain among military helicopter pilots and crew members: a randomized controlled trial protocol

**DOI:** 10.1186/s12891-015-0655-6

**Published:** 2015-08-19

**Authors:** Mike Murray, Britt Lange, Bo Riebeling Nørnberg, Karen Søgaard, Gisela Sjøgaard

**Affiliations:** Department of Sports Science and Clinical Biomechanics, University of Southern Denmark, Odense M, Denmark; Department of Anesthesia and Intensive Care Medicine, Odense University Hospital, Odense C, Denmark; Royal Danish Air Force, Air Force Staff, Defence Command Denmark, Karup J, Denmark

## Abstract

**Background:**

Flight-related neck/shoulder pain is frequent among military helicopter pilots and crew members. With a lifetime prevalence of 81 % for pilots and 84 % for crew members, the prevalence of neck pain is considered high compared to the general population. The aim of this study was to investigate whether a specifically tailored exercise intervention would reduce the prevalence and incidence rate of neck/shoulder pain among helicopter pilots and crew members.

**Method:**

This study used a prospective, parallel group, single blinded, randomized controlled design. Participants were military helicopter pilots and crew members recruited from the Royal Danish Air Force. Inclusion criteria were: 1) employed within the Royal Danish Air Force as a helicopter pilot or onboard crew member (technician, systems-operator, tactical helicopter observer and/or navigator), 2) maintaining operational flight status at enrollment, and 3) operational flying within the previous 6 months. Primary outcome was change in neck and shoulder pain assessed by 1) a modified version of the “Standardized Nordic questionnaire for the analysis of musculoskeletal symptoms” and by 2) pressure pain threshold measurements. Secondary outcomes included: postural balance, strength, stability, and rate of force development for neck and shoulder muscles. Measurements at baseline and follow-up were conducted at four air force bases in Denmark. Sixty-nine participants were individually randomized to either a training group (TG) or a reference group (RG). Participants in the TG performed 20-weeks of physical exercise training divided into sessions of 3 × 20 min per week. Training was completed within working hours and consisted of specific exercise training for the neck and shoulder muscles based on the principles of “Intelligent Physical Exercise Training”. The RG received no training.

**Discussion:**

In spite of the high prevalence of flight related neck/shoulder pain among military helicopter pilots and crew members there are currently no evidence based guidelines for the prevention or clinical handling of neck pain among these occupational groups. Results from this study may therefore be beneficial for future establishment of such guidelines.

**Trial registration:**

Ethical committee of Southern Denmark (S-20120121) 29 August, 2012.

Clinical Trail Registration (NCT01926262) 16 August, 2013.

## Background

Flight related neck pain is frequent among military helicopter pilots and crew members [1, 2]. The 3-month prevalence of neck pain among helicopter pilots is 57 % with approximately 30 % of the cohort reporting recurrent pain episodes [1]. With an lifetime prevalence of 81 % for pilots and 84 % for crew members [3] the prevalence of neck pain is considered high in this occupational group compared to the general population [4].

Helicopter pilots and crew members experience acute, transient, and chronic neck pain related to flight operations [3]. Besides having individual health consequences, neck pain located in the cervical region has been found to influence on: motor control [5, 6], concentration level [7], and postural stability [8], and may therefore have an impact on flight safety [3]. Severe episodes of pain may result in grounding and in rare cases permanent loss of operational flight status, leading to an increase in costs for the Air Force due to loss of manpower and litigation [9, 10].

Flight related neck pain may be described as etiologically non-specific pain. The mechanism is thought to be multifactorial and no obvious pathological mechanism has been identified [9]. However, a number of factors have been hypothesized as possible contributing factors, such as unfavorable and static sitting posture during flight [7, 11], exposure to low-frequency vibrations of high amplitude [12], individual physiological and biological characteristics [13], and prolonged loading of the cervical spine due to the use of a flight helmet and helmet mounted devices such as Night Vision Goggles (NVG) [14, 15].

For more than a decade NVG have provided an advantage to the operational effectiveness of military helicopter flight during low light conditions [16]. However, the weight of a flight helmet with additional NVG may increase the biomechanical stress on the cervical spine, especially during unfavorable head positions [14, 17]. Previous studies have found pronounced weakness and fatigue in the deep segmental neck muscles of helicopter pilots [18] in line with that in patients with chronic neck pain [19]. Excessive loading of the cervical musculature could therefore potentially lead to changes in the neuromuscular function and subsequently lead to neck pain [18]. Physical exercise training was previously found to be effective in significantly reducing flight related neck pain among fighter pilots [20]. However, in spite of a growing level of concern within the operational communities, less research has been conducted regarding specific exercise training for helicopter pilots and crew members [3, 21]. Evidence based guidelines in the prevention and treatment of flight related neck pain among helicopter pilots and crew members are therefore warranted.

### Objective and hypotheses

The primary objective of this study was to design a specifically tailored Intelligent Physical Exercise Training (IPET) program [22] targeting the neck and shoulder muscles, and test if the training could reduce and/or prevent neck/shoulder pain among military helicopter pilots and crew members. Our hypothesis was that IPET would significantly decrease the prevalence and incidence rate of flight related neck/shoulder pain among military helicopter pilots and crew members. The secondary objective was to identify risk indicators for attracting neck pain, such as muscle strength, steadiness, and body balance.

## Methods and design

### Study design

The study design was a 20-week prospective, parallel group, single blinded, randomized controlled trial. The trial was conducted in Denmark from November 2013 to April 2014. Participants were military helicopter pilots and crew members recruited from the Royal Danish Air Force (RDAF). All participants gave their written consent before participation. The trial was approved by the local Ethics Committee of Southern Denmark (S-20120121) and qualified for registration in ClinicalTrails.gov (NCT01926262).

### Participants

**Fig. 1:**
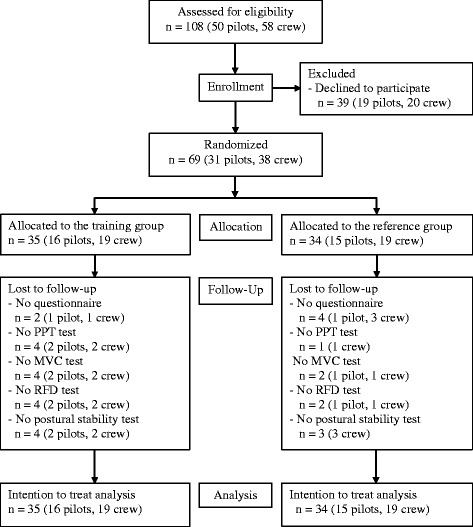
Flow chart. Flow chart of the recruitment of participants

One-hundred and eight pilots and crew members (50 pilots and 58 crew) from two different helicopter squadrons were invited to participate in the present study. All invited participants were informed about the project at briefings within the squadrons as well as by email and telephone. Sixty-nine persons accepted the invitation. Of these 31 were pilots (2 female and 29 male) and 38 were crew members (all male). Inclusion criteria were: 1) employed within the Royal Danish Air Force (RDAF) as a helicopter pilot or onboard crewmember (technician, systems operator, tactical helicopter observer and/or navigator), 2) maintaining operational flight status at enrollment, 3) operational flying within the previous 6 months. Exclusion criteria were: having participated in a training intervention within the last 12 months. Flow of participants is depicted in Fig. 1.

### Procedure of randomization

The 69 participants were assigned a random identification number given to them by an authorized person with no relation to the study. This procedure was concealed from the study investigators to blind randomization and processing of data. After baseline testing, participants were randomized with a ratio of 1:1 to a training group (TG) or a reference group (RG). To ensure comparability between groups, we stratified 67 Danish male helicopter personnel according to the following nested criteria: squadron, job in terms of pilot or non-pilot, age (< or ≥ 40 years of age), and flying experience at the time point of enrollment (< or ≥ than 2500 h). Thereby, theoretically, 16 strata were formed to achieve the above-mentioned balance and de facto pilots were represented in 12 of the strata due to the number of participants from one of the two squadrons being quite small not having participants represented in all eight categories. Two female pilots formed their own strata, resulting in a total of 13 strata that were numbered consecutively. All numbers assigned to the pilots within each stratum were drawn from an opaque, tossed bag. Alternately, the first number in the first strata was allocated to either the TG or RG depending on the flip of a coin. The first number in the second strata was allocated to the opposite group, compared to the last number in the previous strata, and so forth. Thus, all numbers had the same chance of being allocated to the TG or RG. The randomization was carried out by a blinded custodian (last author) using the numbers assigned to the participants. The project leader was not involved in the randomization procedure.

### The training intervention

Participants in the TG were allocated to 20 weeks of physical exercise training, divided into three training sessions of 20 min per week. Training was based on self-management education and was to be performed during working hours. The training program was composed of ten specifically tailored training exercises targeting the neck and shoulder muscles. The specific training program was evidence-based and designed by an interdisciplinary team of sports exercise training specialists, physical therapists, doctors and chiropractors. A detailed description of the individual exercises can be found below. Additional video material is available online [23].

### Conditioning exercises for the neck

**Fig. 2:**
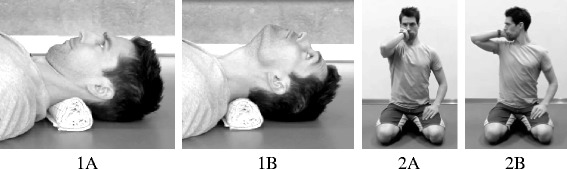
Conditioning exercises for the neck. Conditioning exercises for the deep cervical flexors (Exercise 1 **a** + **b**) and co-contraction between cervical flexor and extensor muscles of the neck (Exercise 2 **a** + **b**)

Based on current literature emphasizing pronounced weakness and fatigue in the deep neck muscles in patients with chronic neck pain, each training session was initiated with conditioning exercises for activation of the deep cervical flexors [19, 24]. The first exercise (Fig. 2, Exercise 1) was performed from a supine position with the back of the head resting on the floor. Participants were instructed to perform an upper cervical spine extension, moving the head backwards in a cephalic direction. When fully extended, the head was returned performing an upper cervical spine flexion in a caudal direction. Participants were instructed to focus on slow and controlled movements and palpate their neck during the exercise to secure that the superficial neck muscles were relaxed. During the first weeks of training a towel was placed underneath the neck for support. As participants progressed, they were instructed to gradually reduce the height of the towel, thereby increasing the training intensity. Eventually participants were instructed to elevate their head a few millimeters above the floor during the exercise [20, 25]. An additional second exercise was introduced at week six. This exercise was designed to train the co-contraction of cervical flexors and extensor muscles (Fig. 2, Exercise 2). The exercise was performed seated with the head held in an anatomical neutral position and one hand placed on the side of the head (right hand for right side rotation and left hand for left side rotation). Participants were instructed to rotate their head against a gentle pressure created by the hand. The exercise was done for both the right and left side. Intensity was gradually increased by creating additional resistance with the hand [20]. The two conditioning exercises were also used for warming up the neck muscles.

### Training exercises for the neck

**Fig. 3:**
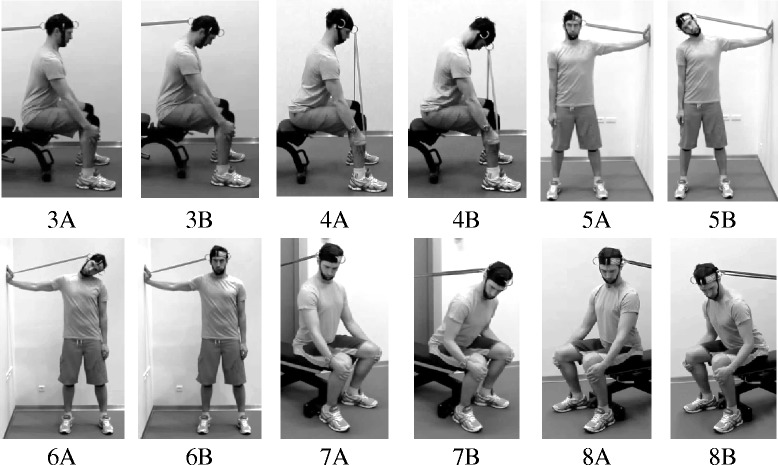
Training exercises for the neck. Training exercises for primary neck muscles during flexion (Exercise 3 **a** + **b**), extension (Exercise 4 **a** + **b**), lateral flexion to the right side (Exercise 5 **a** + **b**) and left side (Exercise 6 **a** + **b**), flexion/rotation to the right side (Exercise 7 **a** + **b**) and left side (Exercise 8 **a** + **b**)

The training program included four training exercises for the prime movers of the neck during cervical flexion, extension and lateral flexion. Exercises were performed with a head harness (Neck Flex, Gonzo Companies, USA) using different color-coded elastic resistance bands (Thera-Band®, The Hygenic Corporation, USA). Cervical flexion was performed seated. Participants were instructed to keep a straight back, position their head in an anatomically neutral position and lean the trunk forward (~20-30°). Arms were held strait with the hands placed underneath the knees. A Thera-Band was stretched between a door anchor and the back of the head harness. During the exercise, participants performed a low cervical spine flexion (against resistance) followed by a low cervical spine extension (Fig. 3, Exercise 3). During neck extension, participants were positioned in the same way as during neck flexion, but the Thera-Band was stretched between the hands and front of the head harness. The exercise was performed with a low cervical spine flexion followed by a low cervical spine extension (against resistance) (Fig. 3, Exercise 4). Lateral flexion was performed standing erect with the head in an anatomically neutral position. One hand was placed horizontally against a wall and a Thera-Band was stretched between the hand and side of the head harness. The exercise was performed with a low lateral spine flexion followed by a low lateral spine extension (against resistance). The exercise was performed for the right (Fig. 3, Exercise 5) and left side (Fig. 3, Exercise 6), respectively. An additional exercise was introduced after six weeks of training simulating the flexed and rotated positioning of pilots and crew members during actual flight. The exercise was performed seated with a straight back and trunk leaned forward (~20°). The head was held in an anatomically neutral position and rotated approximately 45° degrees to either the right or left side. A Thera-Band was stretched between the head harness and a door anchor. Keeping a static upper body, the hips were flexed and the body flexed (against resistance) followed by an extension. The exercise was performed to the right (Fig. 3, Exercise 7) and left side (Fig. 3, Exercise 8).

### Training exercises for the shoulders

**Fig. 4:**
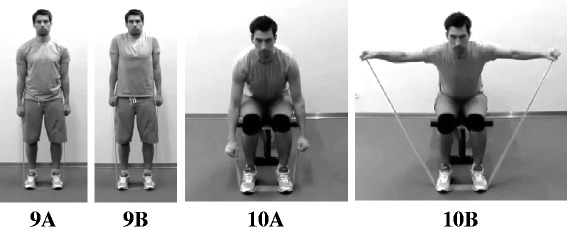
Training exercises for the shoulders. Training exercises for muscles in the shoulder girdle: shrugs (Exercise 9 **a** + **b**) and reverse flies (Exercise 10 **a** + **b**)

The training program included two exercises for muscles in the shoulder girdle. Shrugs were performed standing erect with arms placed along the side of the body and shoulders relaxed. The head was held in an anatomically neutral position with eyes looking straight forward. During the movement, shoulders were elevated as high as possible toward the ears and lowered again [26] (Fig. 4, Exercise 9). Reverse flies were performed seated. Participants were instructed to hold their back straight, position their head in an anatomically neutral position, lean the trunk forward (~20-30°), and place both arms pointing towards the floor. Elbows were kept in a static and slightly flexed position (~5°). During the exercise both arms were raised toward a horizontal level and lowered again [26] (Fig. 4, Exercise 10). The two exercises were previously found to increase strength/endurance and reduce neck/shoulder pain among office workers [27, 28].

The training intervention was based on the principles of Intelligent Physical Exercise Training (IPET). In doing so, the intervention was designed according to: 1) the physiological capacity of participants relative to the occupational exposure, 2) tailoring training exercises to meet individual capacities and disorders, 3) tailoring the intervention to be motivational for participants, and 4) tailoring the intervention to be cost-effective [22]. Tailored workplace exercise protocols have been suggested to be of greater effect, as compared to non-tailored exercise protocols [29, 30]. To allow for a training stimulus that was persistently challenging and effective the training program was designed using recommendations from the American College of Sport Science regarding resistance training for moderately trained adults [31]. The training program focused on improving coordination and increasing endurance and strength in the neck and shoulder muscles using progressive overload. To facilitate strength, but also local muscular endurance, intensity of training ranged between 12–20 repetitions maximum (RM) encompassing 2–4 sets per training session. The training program was design with systematic variation in intensity and volume based on undulating (nonlinear) periodization [31]. The fairly high amount of repetitions would allow for long-duration sets with a high muscle time under tension. With sufficient loading this may increase muscular fatigue that has been found important for stimulating enhancements in local muscular endurance [32]. All exercises were performed with slow velocity during moderate repetition sets (12–15 reps) and with moderate velocity during high repetition sets (15–20 reps) [31]. To further enhance the component of local muscle endurance, participants were instructed to minimize rest periods to 1–2 min during high repetition sets and approximately 1 min during moderate repetition sets [31]. A summation of the total amount of repetitions per exercise per week during the 20 week intervention period is illustrated in Table 1. To allow sufficient time for recovery and reduce the probability of overtraining strength/endurance exercises for the neck and shoulders (Exercise 3 – 10) were performed in an alternating system. Thus, no strength/endurance exercise was performed on two consecutive days. The RG received no specific neck/shoulder training and was told to continue their regular training routines.

**Table 1:** Progression schedule

Week	Exe	Exe	Exe	Exe	Exe	Exe	Exe	Exe	Exe
	1	2	10	4	3	7 + 8	9	6	5
1	6x15	-	5x18-20	5x18-20	5x18-20	-	2x20	2x20	2x20
2	6x15	-	3x18	3x18	3x18	-	6x18	6x18	6x18
3	6x15	-	6x15-18	6x18	6x15-18	-	3x18	3x18	3x18
4	6x15	-	3x18	3x15	3x18	-	6x15-18	6x15-18	6x15-18
5	6x15	-	6x15	6x15	6x15	-	3x15	3x15	3x15
6	6x15	6x15	3x18	3x15	3x18	-	6x15	6x15-18	6x15
7	6x15	6x15	6x15	7x15-18	4x15	2x15	3x18	3x15	3x18
8	6x15	6x15	4x15	3x15	-	2x15	7x15	7x15	8x15
9	6x15	6x15	8x15	8x15	3x15	2x15	4x15	4x15	3x15
10	6x15	6x15	3x15	4x15	3x15	-	8x15	7x15	8x15
11	6x15	6x15	8x12-15	7x15	4x15	2x15	3x15	4x15	3x15
12	6x15	6x15	4x15	4x12	-	2x15	8x12-15	8x12-15	8x15
13	6x15	6x15	8x12-15	8x12-15	4x12	2x12	4x15	4x15	4x12
14	6x15	6x15	3x15	4x12	3x15	-	8x12	7x12-15	8x12
15	6x15	6x15	7x12	7x12-15	4x12	2x15	3x15	4x12	3x15
16	6x15	6x15	4x12	3x12	-	2x15	7x12	7x12	8x12
17	6x15	6x15	8x12	8x12	3x12	2x12	4x12	4x12	3x12
18	6x15	6x15	4x12	3x12	3x12	-	7x12	8x12	7x12
19	6x15	6x15	7x12	7x12	3x12	2x12	4x12	3x12	4x12
20	6x15	6x15	3x12	4x12	-	2x12	7x12	7x12	7x12
Total	1800	1350	1477	1477	904	612	1477	1477	1477

Total number of sets per week and intensities during the 20-week intervention period, for each of the 10 training exercises (Exe). Exercise 1 and 2 are given as number of repetitions. Exercise 3 to 10 are given as repetitions maximum (RM). For instance 6x15-18 RM should be read “6 sets with loadings in the range of 15–18 RM”. The range indicates that there is an undulation in load during that week for this exercise. Exercise 7 and 8 was performed for both right and left side

### Training equipment

Participants in the TG received a personal training bag including: a head harness, Thera-Band resistance bands in 6 color-coded levels of resistance (red, green, blue, black, silver and gold), one set of Thera-Band exercise handles, and one TheraBand door anchor. To secure that training could be done “whenever and wherever” the training program was designed using only light, portable, and robust training equipment. Each participant received a training manual with information and pictures of the different training exercises. The manual was supplemented with online access to a home page with training videos for each exercise.

### Training diary

The training diary included 20 pages (one page for every week of training). Each page was divided into three training sessions and displayed the scheduled training exercises and training intensity. Before and after training participants were asked to fill in the date and place of training. They were also asked to rate their perceived intensity of pain in the neck and shoulders. Pain was rated on a ten point scale from 0 to 9 (0 = no pain, 9 = worst imaginable pain). The training diary included five test sessions, one session every 4 weeks. Participants were instructed to use the test sessions to identify the correct training intensity in the different training exercises. If a participant was able to perform 1–2 repetitions above the specified RM number in the training diary, the training load was to be increased using three steps: 1) first the elastic band was shortened equal to “a hand width”, 2) if this was not sufficient, the elastic band was to be shortened again by one “hand width”, 3) if intensity was still too low, the participant was instructed to change to another elastic band in accordance with the elastic band color code. When the right load was found, the color of the resistance band and hand width position was written in the training diary. In accordance with current approaches to exercise training for the clinical management of spinal pain, it was underlined that participants should keep in mind that training exercises should always be performed with correct technique and controlled movement before increasing training intensity [33]. If a participant in the TG experienced neck, shoulder or back pain that might influence their ability to train, a reduced training program was initiated by the primary investigator. Participants were told only to train the two conditioning exercises used for warming up the neck musculature and training the deep cervical flexors (exercise 1 and 2). Intensity of training was then gradually increased until the individual participant was able to follow the full training program again.

### Outcome measures

The primary outcome of the present randomized controlled trail was change in neck and shoulder pain following the 20 week exercise training intervention. The primary outcome was assessed using a modified version of the “Standardized Nordic questionnaire for the analysis of musculoskeletal symptoms” [34], and by Pressure Pain Threshold (PPT) measurements. Secondary outcomes included: postural balance, strength, stability and rate of force development (RFD) measures for the neck and shoulder muscles.

### Self-reported measures

We applied an online based questionnaire at baseline and after the 20-week intervention period. The questionnaire included: socio-demographic measures (e.g. sex, age, height, weight, family and household configuration, education, financial status), general health and lifestyle (SF-36) [35], work related questions (e.g. flight hours in high performance aircrafts, other aircrafts and helicopters, flight hours with NVG equipment, work schedule, and work hours), work posture, musculoskeletal symptoms (Standardized Nordic questionnaire for the analysis of musculoskeletal symptoms) [34], physical resources [36], level of physical activity (international physical activity questionnaire, IPAQ) [37], workability [38], self-efficacy [39], psychosocial work environment [40], sickness absence [41], work limitations questionnaire [42], and pain perception (Fear-Avoidance Beliefs questionnaire (FABQ) [43]. The main questions are descripted in detail below.

Musculoskeletal symptoms were assessed, for each body region [neck, right/left shoulder, right/left elbow, upper back, lower back, hips, knees and feet] by: 1) The number of days with pain or complaints in [body region] during the last 12 months. Possible answers were: 0 days, 1 – 7 days, 8 – 30 days, 31–90 days, > 90 days, or every day. 2) Inability to perform working tasks due to complaints in [body region] within the last 3 months. Possible answers were: yes or no, and 3) Pain intensity in [body region] within the last 3 months as well as the last 7 days depicted on an 11 point numeric box scale. Possible answers were from 0 = no pain, to 10 = worst possible pain imaginable. Finally, when relevant, the location (body region neck/shoulders) of the most frequent pain episode was recorded. Possible answers were: right side, left side or both sides. All questions were accompanied by chart illustrations of the specific body region in focus.

### Objective measures

Objective measurements were performed at four airbases in Denmark using a flight hangar as the setting for data collection. All measurements were performed by either biomedical laboratory technicians or Master students in sports and health science from the University of Southern Denmark. The test staff was blinded to participants’ group allocation.

Anthropometric measurements included height, seated height, neck circumference, hip/waist ratio, and a Bio-Electrical Impedance Analysis (BIA) (e.g. weight, impedance, fat %, fat free mass, muscle mass, body mass index) (Body Composition Analyzer, SC-331S, Tanita Corporation of America, USA).

Muscular pain level, measured as PPT was assessed bilaterally for the trapezius m. (20 % medial to half the distance between the lateral edge of the acromion and seventh cervical vertebrae) [44], the upper neck muscles (2 cm lateral from the vertical line of the axis in level with the fourth cervical vertebrae) [45, 46], and the anterior tibialis m. as the point of reference [47]. PPT points were marked with a pen. A handheld electronic pressure algometer (Type II Algometor, Somedic Production AB, Sweden) was used, which was pistol-shaped with a pressure-sensitive strain gauge at the tip. The contact area had a diameter of 1 cm^2^ with a thin rubber layer to minimize skin irritation. Compression pressure was applied perpendicularly against the skin with a rate of 20 kPa/s. A digital display on the pressure algometer was used to keep the rate of pressure stable. All PPT measurements were performed three times following a fixed order starting with the right trapezius m., and then left trapezius m., right side of the upper neck muscles, left side of the upper neck muscles, and right tibialis anterior. An intercept of approximately 1 min was given between the three measurements at each PPT point. Each subject was instructed to immediately press a hand held button when the sensation of “pressure” changed to “pain”, at which point compression was stopped and the pressure was released [48]. The maximum applied pressure registered was recorded before resetting the algometer. A maximal pressure of 1000 kPa was allowed for trapezius m. and tibialis anterior m. and 700 kPa for the upper neck muscles. The algometer was calibrated before the beginning of each test using a load of 100 kPa.

Postural stability was measured in five scenarios [8]: 1) One test in the “Romberg position” with feet together, arms crossed in front of the chest and eyes closed. 2) Three repetitions of standing on the dominant leg with the non-dominant leg resting on the medial malleolus of dominant leg, arms crossed in front of the chest and eyes open. 3) Three repetitions of standing on the dominant leg with the non-dominant leg rested on the medial malleolus of dominant leg, arms crossed in front of the chest and eyes closed. 4) Three repetitions of a perturbation test, standing with feet together and eyes open, shoulders flexed in the horizontal plane and elbows slightly flexed, holding a stick (0.73 m) with hands shoulder width apart. A 2.0 kg load was connected to the stick by an electromagnet just before initiation of each perturbation test. The load was released randomly between 5–15 s after start by an electromagnet switch controlled by a computer program. 5) Three repetitions of a perturbation test on a 3.8 cm foam surface, standing with feet together and eyes open. Before each test, the subject was told to focus on a black spot placed 3 m away in eye height. When performing the tests with eyes closed the participant was told to keep focus on the spot while the investigator counted “3-2-1-now”. On the command “now” the participant was told to “close the eyes and stand as still as possible”. Tests were performed without shoes and socks. Participants were instructed to “stand as still as possible” during all tests. Each test lasted 30 s with a pause in between. The tests were performed on a static force platform (AMTI, OR6-7-1000, Watertown, USA). The signal was amplified with a gain of 2000 (AMTI, MSA-6, Watertown, USA). The signal was transformed (National Instrument Corporation, SC-2345, USA) and sampled at 125 Hz using a 16 bit analog-to-digital converter (National Instrument Corporation, DAQCard™ - 6036E, USA). Data was stored on a portable laptop using custom made software and saved for later analysis.

Maximal Voluntary Contraction (MVC) was performed during shoulder abduction and shoulder elevation using a standardized setup and procedure [49]. During shoulder abduction, the participant was seated in an adjustable chair. Arms were held close to the body and elbows were flexed 90°. Two adjustable force dynamometers (load cell, KIS-2, 2kN, Vishay Nobel, Vishay Precision Group, USA) were placed 1 cm above the lateral epicondyle. The lever arm was measured as the distance from the lateral edge of acromion to the transducers. The participant was told to increase the force gradually, reaching MVC within 5 s and hold the force for 2 s before slowly reducing force again. During shoulder elevation, the force transducers were placed on both shoulders one cm medial to the lateral edge of the acromion. The lever arm was measured from the seventh cervical vertebral (C7) to the transducers. A minimum of three MVC tests were performed. If the result for the third MVC was ≥5 % compared to the first or second MVC, another MVC trial was performed. A maximum of five MVC´s was allowed for each test. All tests were performed with verbal encouragement. Force was amplified with a gain of 100 (National Instruments Corporation, Full bridge amplifier, SCC-SG24, USA), and sampled at 100 Hz using a 16-bit analog-digital converter (National Instruments Corporation, DAQ Card ™ - 6034E, USA). Data was stored as torque on a computer and saved for later analysis.

Steadiness was measured after shoulder elevation using the same setup. The two force dynamometers were lowered to secure a constant pressure of 2–3 kg on each shoulder. A computer monitor was placed in front of the subject for visual feedback. The monitor illustrated a horizontal line corresponding to 30 % MVC during shoulder elevation. Force was illustrated as a black cursor on the monitor that could be controlled by increasing or decreasing pressure against the force transducers. Subjects had 5 s in the beginning of the test to elevate their shoulders and achieve a pressure corresponding to the 30 % MVC line. All subjects were told to place the black cursor “as close as possible to the horizontal line and keep it as stable as possible” during the 30 s test. All subjects were told to elevate both shoulders, but only force input from the dominate shoulder was recorded.

Rate of force development (RFD) was measured during shoulder elevation, cervical flexion and cervical extension. Instructions for the RFD tests were: “on the command “3-2-1” you must apply a slight pressure against the force transducer and on NOW…press as hard and fast as possible. You must keep the pressure for a second and then slowly relax again”. During RFD for shoulder elevation subjects were positioned as during MVC measurements. During neck flexion, subjects were positioned seated in an upright position. Arms were placed along the side and feet on the floor. The head and neck was held in an anatomical neutral position and a force dynamometer was positioned just above the eyebrows. Before testing, subjects were strapped firmly into place using belts. During neck extension, subjects were positioned similar to the position during neck flexion, but with the back against the experimental setup. The head and neck was held in an anatomical neutral position and a force dynamometer was placed just above external occipital protuberance. The distance from C7 to the center of the transducer was measured for analyzing torque during neck flexion and extension. Rate of force development was determined as the rate of torque development, and maximal muscle strength was determined as the peak torque. Force was amplified with a gain of 100 (National Instruments Corporation, Full bridge amplifier, SCC-SG24, USA) and sampled at 1000 Hz using a 16-bit analog-digital converter (National Instruments Corporation, DAQ Card ™ - 6034E, USA). Data was stored on a computer and saved for later analysis.

### Statistics

Power analysis showed that we would need to include 54 participants (27 experimental subjects and 27 control subjects) in this study. The calculation was based on the finding that a change of 1 measured on a 11 point numeric box scale, is considered the minimum clinically significant difference regarding change in pain [50]. We also used results on pain intensity from a previous study that found the response within each subject group to be normally distributed with a standard deviation of 1.5 [20]. With a power set at 0.8 and a probability of a type I error p < 0.05, we will be able to detect a true difference in mean response of pain between experimental and control subjects equal to ± 1.2 measured on an 11 point numeric box scale. Allowing for a 10 % loss to follow-up, the total number of participants was decided to be 64. All randomized participants were included in the data analysis according to the intention-to-treat principle [51]. Missing data was to be imputed subsequently using a sensitivity analysis in order to explore the effect of different data imputations. Secondary analysis was to be performed according to the principle of per protocol analysis including only participants who completed the training originally allocated. Regular adherence was to be defined as training between 1–3 times a week during the 20-week intervention period [20]. Data analysis will be performed using Stata statistical software version 13 (StataCorp LP, USA).

## Discussion

Specific strength training has been considered preventive regarding neck pain among military pilots for some time. However, only a few studies have investigated this hypothesis, and until recently no studies had found a significant positive effect as demonstrated for fighter pilots [20]. Due to different types of exposures during flight, results from studies regarding fighter pilots cannot be directly transferred to helicopter pilots and crew members. Also, fighter pilots experience more acute types of neck pain, while helicopter pilots and crew members experience primarily sub-acute and chronic pain [18]. The pain origin is therefore not homogeneous and a preventive and management strategy should be designed differently among the two groups.

Based on our primary outcome investigating whether an exercise training intervention is effective in reducing and preventing pain, we included participants with and without neck pain at baseline. Given the high prevalence of neck pain within this occupational group, illustrated by previous studies [1, 2], the likelihood of new neck pain cases within the intervention period is high. This in turn also rationalizes the inclusion of asymptomatic participants at baseline. Inclusion of asymptomatic participants may, however, attenuate the mean values of reported neck pain within groups. If only pain-cases were enrolled, we would expect the mean intensity of neck pain to be higher. It might be suggested, that the minimal clinically significant difference, measured on a visual analog scale, is not the same across the whole range of the scale, and that a lower mean level of pain intensity could therefore bias the interpretation of our study results. However, it has previously been found, that the minimal clinically significant difference in pain score, measured on a 100 mm visual analogue scale (VAS), did not differ with the severity of pain being experienced [52]. Previously a pain reduction equal to 1.0 measured on a 11 point numeric box scale, has been considered the minimal clinical important change [50].

### Strengths and limitations

This study has several strengths, the first being the use of a prospective, parallel group, single blinded randomized controlled design. Also, an innovative training program which uses evidence-based principles of IPET is used [22]. The training program is designed through an interdisciplinary teamwork resulting in a high quality training program targeting the pain inflicted neck muscles. The cost of the study is kept low using self-administrated training. However, this study also has limitations that need attention. The present study includes participants with and without neck pain. Participants without neck pain at baseline may be less motivated for exercise training compared to participants with neck pain. This may influence compliance and thereby affect our primary outcome. Finally, due to a dynamic work environment there is a risk of contamination between participants within the TG and RG.

## Abbreviations

BIA: Bio-electrical impedance analysis
IPET: Intelligent physical exercise training
MVC: Maximal voluntary contraction
NVG: Night vision goggles
PPT: Pressure pain threshold
RDAF: Royal danish air force
RG: Reference group
RFD: Rate of force development
TG: Training group
VAS: Visual analogue scale
